# Artificial intelligence enabled preliminary diagnosis for COVID-19 from voice cues and questionnairesa)

**DOI:** 10.1121/10.0003434

**Published:** 2021-02-11

**Authors:** Carmi Shimon, Gabi Shafat, Inbal Dangoor, Asher Ben-Shitrit

**Affiliations:** 1 Afeka College of Engineering, Tel Aviv, Israel; 2 Matrix IT Ltd., Herzliya, Israel

## Abstract

The COVID-19 outbreak was announced as a global pandemic by the World Health Organization in March 2020 and has affected a growing number of people in the past few months. In this context, advanced artificial intelligence techniques are brought to the forefront as a response to the ongoing fight toward reducing the impact of this global health crisis. In this study, potential use-cases of intelligent speech analysis for COVID-19 identification are being developed. By analyzing speech recordings from COVID-19 positive and negative patients, we constructed audio- and symptomatic-based models to automatically categorize the health state of patients, whether they are COVID-19 positive or not. For this purpose, many acoustic features were established, and various machine learning algorithms are being utilized. Experiments show that an average accuracy of 80% was obtained estimating COVID-19 positive or negative, derived from multiple cough and vowel /a/ recordings, and an average accuracy of 83% was obtained estimating COVID-19 positive or negative patients by evaluating six symptomatic questions. We hope that this study can foster an extremely fast, low-cost, and convenient way to automatically detect the COVID-19 disease.

## I. INTRODUCTION

Scientists and researchers from a bench of research domains are stepping up in response to the challenges raised by the COVID-19 pandemic and its consequences. Meanwhile, methods and technologies have been designed and investigated to accelerate diagnostic testing speed (Durner *et al.*, 2020).

A lot of data driven efforts have been made regarding to the Covid-19 pandemic. In particular, a number of works have proposed the promotion of sound-based COVID-19 assessment. For instance, in Durner *et al.* (2020), a model based on a convolutional neural network (CNN) was developed to extract visual features from mel-spectrogram images to classify four cough types (COVID-19, pertussis, bronchitis, and normal).

From the perspective of sound analysis, as coronavirus is a respiratory illness, abnormal breathing patterns from patients intuitively might be a potential indicator for diagnosis of sleep quality, anxiety, fatigue (Han *et al.*, 2020), or any other abnormal respiratory activity (Wang *et al.*, 2020). Other research explores the changes in the acoustic parameters of voice in COVID-19 patients (Asiaee *et al.*, 2020). Various typical respiratory symptoms can be observed, from dry cough presented in mild illness to shortness of breath in moderate illness and, further, severe dyspnea, respiratory distress, or tachypnea in severe illness (Cascella *et al.*, 2020).

In this research, we investigate the importance of analyzing voice or speech signals and a short questionnaire regarding this virus pandemic in an empirical manner. As part of an early study on the intelligent analysis of voice cues under COVID-19, a data-driven approach automatically detects the patients' health status. It is our hope that this step can help develop a rapid, inexpensive, and non-invasive way to diagnose the COVID-19 disease and assist medical doctors.

## II. DATA COLLECTION

Since the COVID-19 pandemic is still spreading, data collection and annotation is an ongoing task. At present, data collection is under way worldwide from both infected patients at various stages of the disease and healthy individuals as a control group. Researchers from Vocalis-Health Company,^1^ in a collaboration with the Israeli Defence Forces (IDF),^2^ the Afeka Center for Language Processing (ACLP), and Matrix IT Ltd., have launched a new app to gather voice samples as well as a short query regarding symptoms such as fever, shortness of breath, tiredness, etc. Researchers from Carnegie Mellon University (CMU) have launched a new web page^3^ as a “COVID-19 Voice Detector” to gather voice samples, such as coughs, several vowel sounds, counting up to 20, the alphabet, etc. Nonetheless, currently all these data are not publicly available for research purposes according to the Helsinki Committee. The recordings from CMU are self-recorded, i.e., no medical doctor has confirmed the patient's status. As for the IDF data collection, the process took place in Israel using the Vocalis-Health app. It was supervised by medical doctors who instructed verified COVID-19 positive and negative patients on how to record themselves. At this point, data collection between March 6 and June 13, 2020, is being used and processed. While doctors were making their daily rounds to check the patients at the hospitals, they recorded each patient individually. Data collection from IDF consists of three vowels (/a/, /s/, and /z/), coughs, a short reading passage, and counting from 50 to 80 in the Hebrew language. The focus is on cough and /a/ vowel recordings solely based on experimental results. Furthermore, regarding demographic information from both datasets, two characteristics of the patients were collected: age and gender. A statistical overview of the data is shown in Table I.

**TABLE I.** Distribution of recording segments collected from 130 COVID-19 negative and 69 COVID-19 positive patients, where P stands for positive and N for negative.

Voice cue	COVID-19 P	COVID-19 N	Total recordings
# cough	513	783	1296
# /a/	282	146	428

As of June 13, 2020, the text data collection contains 173 questionnaires from 57 patients, 25 of them COVID-19 positive and 32 negative. Forasmuch as patients stayed at the hospital for more than 1 day, some of them answered the questionnaire multiple times, in particular, 1–16 times. The following six questions were asked in the questionnaire: (i) How bad is your shortness of breath today? (ii) How bad is your cough today? (iii) How bad is your snot today? (iv) Have you measured fever over 37.8 °C today? (v) Is there a change in the sense of smell? (vi) In relation to the earlier days, how do you feel today?

### A. Audio quality

The voice capturing process from both CMU and IDF was done using an application installed in the patient's smartphone, which applied postprocessing to the audio signal and used compression by various vocoders. While for most of the people, this fact will make no difference, this process makes changes in the RAW audio file.

## III. DATA PREPROCESSING

A series of data preprocessing processes were implemented, specifically the following three processes.

### A. Data cleansing

Recordings were made in hospitals, in a noisy environment. Consequently, recordings that contained both noisy background and shorter than 500 ms for vowel /a/, and shorter than 100 ms for coughs were discarded, since some patients were experiencing difficulties pronouncing.

Regarding vowels /a/, 5% of leading and trailing samples were trimmed out to avoid inhale or exhale effects.

### B. Voice activity detection

audacity software was used to segment coughs for each voiced segment in case a recording consisted of multiple coughs in a row.

Following audio-data preprocessing, a total number of 1296 segments for coughs and 428 segments for /a/ vowels existed for audio experiments. Statistics of the distribution are presented in Table I. In total, 1728 audio segments were collected with a sampling rate of 44.1 kHz for further analysis.

### C. Text data quantization

Since the answers for the questionnaire were in words (none, very mild, discomforting, moderate, severe, and very severe), a quantization had to be done, i.e., answers were converted to numbers between 1 and 6 and then normalized using min-max normalization to numbers between 0 and 1.

## IV. FEATURE EXTRACTION

Three acoustic feature sets are considered in this study. First was the Computational Paralinguistics Challenge (ComPARE); specifically, these feature sets were extracted with the openSMILE toolkit (Eyben *et al.*, 2010). Second was a combination of acoustic features extracted from a freely available script and libraries with the open-source software, praat and librosa. The first is a software that analyses, synthesizes, and manipulates speech.^4^ The second is a python library for audio and music analysis (McFee *et al.*, 2015). Another acoustic feature-set is a 1024 embedding feature vector, extracted per utterance using a deep convolutional neural network (D-CNN). The D-CNN model was prior trained for weakly labeled audio by Kumar *et al.* (2018) using the ESC-50 dataset.

The ComPARE feature set is a large-scale brute-force set (Schuller *et al.*, 2013). It contains 6373 static features by computing various statistical functionals over 65 low-level descriptor (LLD) contours, cepstral, prosodic, and voice quality features. For more details, the reader is referred to Schuller *et al.* (2013).

Similar to the large-scale ComPARE set, a smaller feature set is based on praat^4^ and librosa (McFee *et al.*, 2015). It contains 65 static features by computing various statistical functionals over some other LLD. 65 features are carefully selected based on trial and error on COVID-19 positive and negative recordings, using RF tests.

Another feature set applied in this work is based on a D-CNN model (Kumar *et al.*, 2018), which was designed and trained to classify 2000 weakly labeled environmental audio recordings from the ESC-50 dataset. Then Kumar *et al.* (2018) used transfer learning on 50 different classes of speech and environmental sounds where one of them was coughs. The last layers of the D-CNN were the 1024 embedding feature vector and a classification layer. For more details, the reader is referred to Kumar *et al.* (2018). The researchers' aim is COVID-19 identification. An audio recording (cough or /a/) is the input of the neural network, while the output is a 1024 embedding feature vector from the D-CNN model to be used as a feature set.

In addition to the audio recordings, a six value vector, quantized between 0 and 1 was used according to the questionnaire answers from the IDF data collection.

## V. EXPERIMENTS

In this work, the evaluation of the three feature sets is against two classifiers: a support vector machine (SVM) with a radial basis function (RBF) and a random forest (RF). SVMs are known as both linear and non-linear classifiers that map input features into high-dimensional feature spaces, allowing them to find the best separation between classes. RFs are an ensemble learning method for classification and operate by constructing a multitude of decision trees at training time and outputting the class that is the mode of the classes. They both work well for small and large amounts of data, differing from neural networks, which require a large amount of data for training. The two classifiers were implemented in python using the scikit-learn library.^5^ Many parameters were tuned to get the best results, specifically, for the RF classifier, 20 estimators (number of trees) with a maximum depth of 4 for each tree. As for the SVM classifier, considering linear, polynomial, and RBF kernel functions, the most separating kernel was found to be the RBF one. Furthermore, to deal with the imbalanced data during training, a class weighting strategy was employed.

For all experiments in this study, the same train-set and test-set were used. The data were divided as shown in Table II.

**TABLE II.** Train-test partition of the COVID-19 audio data, where P stands for positive and N for negative.

Set	Voice cue	COVID-19 P	COVID-19 N
Train	# cough	27	90
	# /a/	22	23
Test	# cough	10	8
	# /a/	10	9
Total patients	# cough	37	98
	# /a/	32	32

For the examinees evaluated for coughs, the average age was 32.8 ± 12 yrs. For those evaluated for vowel /a/, the average age was 29.6 ± 9 yrs. Notice that the test-set obeys the following requirements:

(1) No overlapped patients (speakers) in train-test-sets,
(2) Only IDF data (COVID-19 verified by medical doctors),
(3) COVID-19 positive-negative balance,
(4) Demographic balance (age and gender).

### A. Majority voting

Since patients provided multiple recordings each day, for several days (varied from patient to patient), our algorithm decision was based on multiple classifications, meaning that a classifier (cough, /a/, or both) evaluated each recording and provided a classification result. Later, a collection of recordings was classified according to the most frequent classification (COVID-19 positive or negative). The following four are the types of recording collection:

(1) Voting per-patient, per recording day using multiple voice cues (multiple classifiers). In other words, for each patient, a classification is based on recordings from a single day.
(2) Voting per-patient, per recording day, per voice cue. In other words, for each patient, a classification is based on recordings from a single day for only one voice cue.
(3) Voting per-patient (all days), per voice cue. In other words, for each patient, a classification is based on all available recordings for only one voice cue.
(4) Voting per-patient (all days), using multiple voice cues (multiple classifiers). In other words, for each patient, a classification is based on all available recordings.

Both COVID-19 positive and negative patients stayed at the hospitals for more than one day and recorded themselves multiple times. This fact allows us to take advantage and use multiple recordings of patients.

A majority threshold value of 60% was determined. If 60% of the recordings were classified as COVID-19 positive, the patient was declared positive.

## VI. RESULTS

In this section, a detailed performance report of the best models, from varied classifiers, for the three selected feature sets, on test-set,^6^ is exhibited and discussed.

### A. Audio results

#### 1. Vowel /a/ results

Handcrafted features, which were extracted using praat and librosa, performed better than the 1024 embedding feature vector and ComPARE feature set, in both RF and SVM classifiers, achieving 0.76 accuracy, 0.67 area under the curve (AUC), and 0.84 F1-score, as presented in Table III.

**TABLE III.** Performance in terms of accuracy, AUC, F1-score, sensitivity, and specificity for two-class COVID-19 classification using RF and SVM classifiers for both **vowel /a/** and **coughs**. Clf, classifier; Acc, accuracy; Sens, sensitivity; Spec, specificity.

Voice cue	Clf	Feature	Acc	AUC	F1	Sens	Spec
/a/	RF	praat + librosa	0.76	0.67	0.84	0.91	0.41
Cough			0.70	0.58	0.80	0.85	0.34
/a/		ComPARE	0.57	0.62	0.63	0.51	0.71
Cough			0.61	0.69	0.65	0.51	0.84
/a/		Embedding	0.73	0.53	0.84	0.98	0.08
Cough			0.73	0.69	0.81	0.82	0.52
/a/	SVM	praat + librosa	0.78	0.64	0.86	0.95	0.36
Cough			0.59	0.63	0.64	0.52	0.76
/a/		ComPARE	0.64	0.57	0.75	0.76	0.35
Cough			0.74	0.60	0.83	0.90	0.35
/a/		Embedding	0.68	0.51	0.80	0.90	0.14
Cough			0.60	0.63	0.67	0.57	0.35

#### 2. Cough results

The 1024 embedding feature vector, which extracted using D-CNN for sound events and scenes (Kumar *et al.*, 2018), performed much better than the other feature sets. Note that the RF classifier performed better than the SVM classifier, achieving 0.73 accuracy, 0.69 AUC, and 0.81 F1-score. The best performance was taken where the highest AUC was obtained, and performance in terms of accuracy and F1-score is given.

### B. Majority voting audio results

COVID-19 is known for its respiratory symptoms [Wang *et al.* (2020) and Cascella *et al.* (2020)], which can be observed, from dry cough presented in mild illness to shortness of breath in moderate illness. As a result, it would be interesting to examine the results on the cough classification task using a majority voting method, especially per-day. Considering the most effective threshold value, a threshold was found based on experiments on the train-set using a wide range, 10%–90%. Eventually, the most effective threshold was set to 60%. Results per-day, per-patient are shown in the first three rows of Table IV, while results per-patient (all recordings from all days) are shown in the last three rows of Table IV. In Table IV, results are boosted by using the majority voting per-day method for both /a/ vowels and coughs, achieving 0.69 and 0.74 AUC scores, respectively. On the contrary, per-patient (all day recordings) results are poorer. A combination of both classifiers and feature sets for /a/ and coughs, per-day, shows the most promising results (Tables V–VII).

**TABLE IV.** Performance in terms of accuracy, AUC, F1-score, sensitivity, and specificity for two-class COVID-19 classification using RF and SVM classifiers followed by majority voting method, for **coughs**, **/a/**, and **coughs + /a/**, per-day and per-patient. Acc, accuracy; Sens, sensitivity; Spec, specificity.

Metric	Voice cue	Acc	AUC	F1	Sens	Spec
Per-day	Cough	0.78	0.74	0.84	0.85	0.65
	/a/	0.75	0.69	0.83	0.85	0.53
	Cough + /a/	0.80	0.74	0.86	0.91	0.56
Per-patient	Cough	0.72	0.70	0.78	0.90	0.50
	/a/	0.58	0.57	0.64	0.70	0.44
	Cough + /a/	0.61	0.59	0.69	0.80	0.37

**TABLE V.** Confusion matrix for all patients, per-day recordings, for **coughs**.

	COVID-19 negative	COVID-19 positive	Total
COVID-19 negative	11	5	16
COVID-19 positive	5	29	34
Total	16	34	50

**TABLE VI.** Confusion matrix for all patients, per-day recordings, for **/a/** vowels.

	COVID-19 negative	COVID-19 positive	Total
COVID-19 negative	9	5	14
COVID-19 positive	4	36	40
Total	13	41	54

**TABLE VII.** Performance in terms of accuracy, AUC, F1-score, sensitivity, and specificity for two-class COVID-19 classification based on a self-report questionnaire. Acc, accuracy; Sens, sensitivity; Spec, specificity.

Classifier	Acc	AUC	F1	Sens	Spec
SVM	0.72	0.76	0.75	0.64	0.88
RF	0.83	0.86	0.86	0.77	0.94

### C. Text results

In total, 173 questionnaires were self-reported, 96 by COVID-19 positive patients and 77 by negative ones. Of the questionnaire answers, as for COVID-19 negative patients, the average answer score is 2.3. As for COVID-19 positive patients, the average answer score is 2.9, which is considerably higher than the score of COVID-19 negative patients, meaning negative patients claim fewer symptoms. Classification results based solely on text are shown in Table VIII.

**TABLE VIII.** Performance in terms of accuracy, AUC, F1-score, sensitivity, and specificity for two-class COVID-19 classification using the best feature sets and classifiers followed by the majority voting method, both for **coughs** and **vowel /a/**, per-day recordings. Acc, accuracy; Sens, sensitivity; Spec, specificity.

Metric	Voice cue	Acc	AUC	F1	Sens	Spec
Per-day	Cough + text	0.80	0.77	0.85	0.85	0.68
	/a/ + text	0.79	0.71	0.85	0.90	0.53

### D. Majority voting audio and text results

Classification tasks based on self-reported answers on the questionnaire are much more accurate than the results based solely on audio recordings. Accordingly, it is recommended to use both types of information, similarly to a medical doctor's diagnosis.

### E. Discussion

In this preliminary study, experiments were carried out based on speech recordings and a self-reported questionnaire, from COVID-19 infected and hospitalised patients. The results have demonstrated the feasibility and effectiveness of audio-and-text-based COVID-19 analysis, specifically in predicting the health status of patients. Nonetheless, there are still many ways to extend the present study for further development. First, the collected dataset is relatively small and lacks patients with other respiratory diseases. Hence, further research must be established to investigate whether the classifier can distinguish between other respiratory diseases and COVID-19. Additionally, we do not know the medical background of the patients, which may bias the classification task. Therefore, the COVID-19 positive and negative terminology is used.

These data collections are still in progress for more comprehensive analysis in the future. Also, given more data, the performance of our models is expected to be further improved and more robust. Moreover, in addition to conventional handcrafted features, deep representation learning algorithms might be explored to learn representative and salient data-driven features for COVID-19 related tasks. In this study, the classification results based on days recording during hospitalisation are much better than the results per-patient (all day recordings), as can be seen in Table IV. We assume these findings are related to the fact that COVID-19 positive individuals are recovering over time; therefore, their respiratory symptoms become less apparent.

## Footnotes

^1^ https://vocalishealth.com/ (Last viewed February 1, 2021).

^2^ https://www.idf.il/en/minisites/idfs-response-to-covid-19/idf-develops-technology-to-battle-covid-19 (Last viewed February 1, 2021).

^3^ https://cvd.lti.cmu.edu/ (Last viewed February 1, 2021).

^4^ https://www.fon.hum.uva.nl/praat/ (Last viewed February 1, 2021).

^5^ https://scikit-learn.org/stable/ (Last viewed February 5, 2021).

^6^ In this experiment, cross-validation was not used.
